# AGE-RELATED VALUE ORIENTATIONS IN LARGE LANGUAGE MODELS (LLMS)

**DOI:** 10.1093/geroni/igae098.3251

**Published:** 2024-12-31

**Authors:** Xin Zhang, Yuanyi Ren, Guojie Song

**Affiliations:** Peking University, Beijing, Beijing, China (People’s Republic); Peking University, Beijing, Beijing, China (People’s Republic); Peking University, Beijing, Beijing, China (People’s Republic)

## Abstract

Large Language Models (LLMs) are transforming diverse fields and gaining increasing influence as human proxies. This development underscores a critical scientific need to evaluate their value orientations and understanding, which are major determinants of individual and collective human behavior. We used a newly developed evaluation pipeline grounded in realistic human-AI interactions to probe age-related value orientations (e.g., hostile and benevolent ageism) of six different LLMs (including GPT-3.5, GPT-4, Llama2-7B, Llama2-70B, Mistral-7B and Mixtral-8x7B). The results indicated that compared with human participants, these Large Language Models exhibited significantly less hostile ageism in terms of both descriptive and prescriptive ageism, while these Large Language Models exhibited a similar level of benevolent ageism to the degree of human participants. Moreover, we also observed a reversed aging stereotype pattern such that in contrast to typical High-Warmth-Low-Competence stereotype according to stereotype content model, Large Language Models exhibited a High-Warmth-High-Competence stereotype on older adults. Further experiment was conducted to examine how such age-related value orientation of LLMs (especially the benevolent ageism and the high-competence stereotype) could influence individuals’ attitudes as well as behavioral tendencies toward older adults. 80 younger adults were randomly assigned to discuss age-related topics with either LLMs or humans, who will present previously generated contents expressing benevolent ageism and high competence stereotype toward older adults. Results indicated that participant’s attitudes (as measured by SCM and AAS) as well as helping behaviors (in terms of SVO and third-party punishment game) toward older adults were most influenced by LLMs compared with humans.

